# Fungal diversity in sediments of the eastern tropical Pacific oxygen minimum zone revealed by metabarcoding

**DOI:** 10.1371/journal.pone.0301605

**Published:** 2024-05-13

**Authors:** Judith Posadas, Patricia Velez, Silvia Pajares, Jaime Gasca-Pineda, Laura Espinosa-Asuar

**Affiliations:** 1 Posgrado en Ciencias del Mar y Limnología, Instituto de Ciencias del Mar y Limnología, Universidad Nacional Autónoma de México, Mexico City, Mexico; 2 Departamento de Botánica, Instituto de Biología, Universidad Nacional Autónoma de México, Mexico City, Mexico; 3 Unidad Académica de Ecología y Biodiversidad Acuática, Instituto de Ciencias del Mar y Limnología, Universidad Nacional Autónoma de México, Mexico City, Mexico; 4 Departamento de Ecología Evolutiva, Instituto de Ecología, Universidad Nacional Autónoma de México, Mexico City, Mexico; 5 Instituto de Ecología, Universidad Nacional Autónoma de México, Mexico City, Mexico; Bristol-Myers Squibb Company, UNITED STATES

## Abstract

Oxygen minimum zones (OMZ) represent ~8% of the ocean, with the Pacific as the largest and top expanding area. These regions influence marine ecosystems, promoting anaerobic microbial communities. Nevertheless, only a fraction of microbial diversity has been studied, with fungi being the less explored component. So, herein we analyzed fungal diversity patterns in surface and subsurface sediments along a bathymetric transect using metabarcoding of the ITS1 region in the OMZ of the Mexican Pacific off Mazatlán. We identified 353 amplicon sequence variants (ASV), within the *Ascomycota*, *Basidiomycota*, and *Rozellomycota*. Spatial patterns evidenced higher alpha diversity in nearshore and subsurface subsamples, probably due to temporal fluctuations in organic matter inputs. Small-scale heterogeneity characterized the community with the majority of ASV (269 ASV) occurring in a single subsample, hinting at the influence of local biogeochemical conditions. This baseline data evidenced a remarkable fungal diversity presenting high variation along a bathymetric and vertical transects.

## 1. Introduction

Oxygen minimum zones (OMZ) are oceanic regions with very low oxygen (O_2_) concentrations (<22 μmol kg^-1^; [1]). They are estimated to cover ~8% of the oceanic surface and ~1% of its volume, with the lowest O_2_ concentrations occurring in the Pacific and Indian Oceans [2, 3]. Despite their low coverage, OMZ are a critical source of nitrous oxide (producing about 20% of total global emissions) and are responsible for up to 50% of the oceanic fixed-nitrogen loss, releasing nitrous oxide (ozone-destroying potent greenhouse gas) into the atmosphere, and limiting global productivity with the consequent feedback on the carbon cycle [4, 5]. These regions usually occur as a consequence of the joint effect of physical, chemical, and biological processes such as thermal stratification, poor ocean circulation, limited air-sea O_2_ exchange, low solubility of O_2_ at high temperatures, and the upwelling of nutrient-rich waters to the surface that drives biological productivity and O_2_ consumption [2, 6, 7]. Oxygen loss in the open ocean has increased over the past 30–50 years, mainly due to anthropogenic climate change [1, 8–10]. This expansion implies adverse changes to marine ecosystems, including the benthic community since 0.3% of the global seafloor is intercepted by OMZ waters [6, 8, 9]. Therefore, the analysis of sediment biota is essential for OMZ monitoring.

The OMZ are dominated by microbes [10], because most the multicellular organisms are adversely affected by the O_2_ deficiency that exerts strong selective pressures [11]. On the one hand, several studies have focused on the evaluation of prokaryotic community composition and its contribution to nitrogen, sulfur, and carbon cycles via chemosynthesis [10, 12–14]. On the other hand, microeukaryotes have shown specialized lifestyles, contributing to carbon and nitrogen cycles via interactions with prokaryotes and denitrification processes, respectively [15–19]. Community composition in these groups varies according to site conditions. Nonetheless, the diversity of microeukaryotes such as fungi has been poorly studied, despite their large contribution to diversity in OMZ [20–22]. In early culture-based investigations, the copious occurrence of fungi in the sediments of OMZ has been registered [20, 21]. The obtained isolates have shown the ability to grow under anoxic conditions in the laboratory [23], as well as an active role in oceanic nitrogen and carbon cycles [24, 25]. Nevertheless, the identification of some fungal groups remains a challenge, since several taxa are not easily cultured. In fact, it has been estimated that fungal diversity detected with culture-independent approaches is 8.8 times greater than the diversity assessed with culture-dependent approaches [26].

Environmental DNA studies, including high-throughput Illumina sequencing of the internal-transcribed spacer (ITS) region, have revealed the presence of abundant uncultured fungal phylotypes and environmental clusters in the OMZ of the Indian, Atlantic and Pacific Oceans within the *Ascomycota*, such as the *Pezizomycotina* clone group (PCG) and the deep-sea fungal group-1 (DSF-Group1); and the *Basidiomycota*, including the hydrothermal and/or anaerobic fungal group (Hy-An Group; [20, 27, 28]. In addition, this approach has allowed the thorough description of fungal communities, evidencing the dominance of the *Ascomycota*, *Basidiomycota* [20–24], and basal fungal lineages [27, 29]. These findings suggest that OMZ harbor a large proportion of fungal taxa that remain to be described.

Fungal communities in surface sediments sampled along a bathymetric transect have generally shown large variations in their composition [29–31]; in contrast, surface sediments along the South China Sea showed similarity to each other [32]. Overall, fungal diversity figures are higher at nearshore than offshore benthic sites [33, 34], perhaps because of the sediment source, geographic distance, or site-specific environmental factors [32, 33, 35, 36].

The occurrence of fungi has been also recorded in subsurface sediments, such as those in the Canterbury Basin [37, 38], Suruga-Bay [39], and Peru Margin [40]. In these sediments, fungi have been considered the dominant eukaryotic kingdom [30] and the third most abundant microbial component (after Bacteria and Archaea; [38]). Within vertical gradients, fungal composition can be highly variable at the small-scale (<100 cm below seafloor; [29, 41]) and the large-scale (>100 cm below seafloor; [30, 32, 38] by the possible influence of site-specific conditions. However, to the extent of our knowledge, fungal diversity in the Mexican OMZ remains unexplored, resulting in a limited understanding of the community structure and its importance in the ecosystem. Herein, we used high throughput sequencing of the ITS1 region to analyze fungal community composition and diversity in surface (0–10 cm) and subsurface (10–20 cm) sediments collected in four stations along a bathymetric transect (32–705 m) in the OMZ of the Mexican Pacific off the Port of Mazatlán. We hypothesize that: 1) sediments in this OMZ will harbor a high fungal diversity dominated by the *Ascomycota*, 2) the highest diversity levels will be observed in nearshore stations, and 3) community composition will vary at the small-scale.

## 2. Materials and methods

### 2.1. Study area, sampling, and chemical analyses

**Fig 1:**
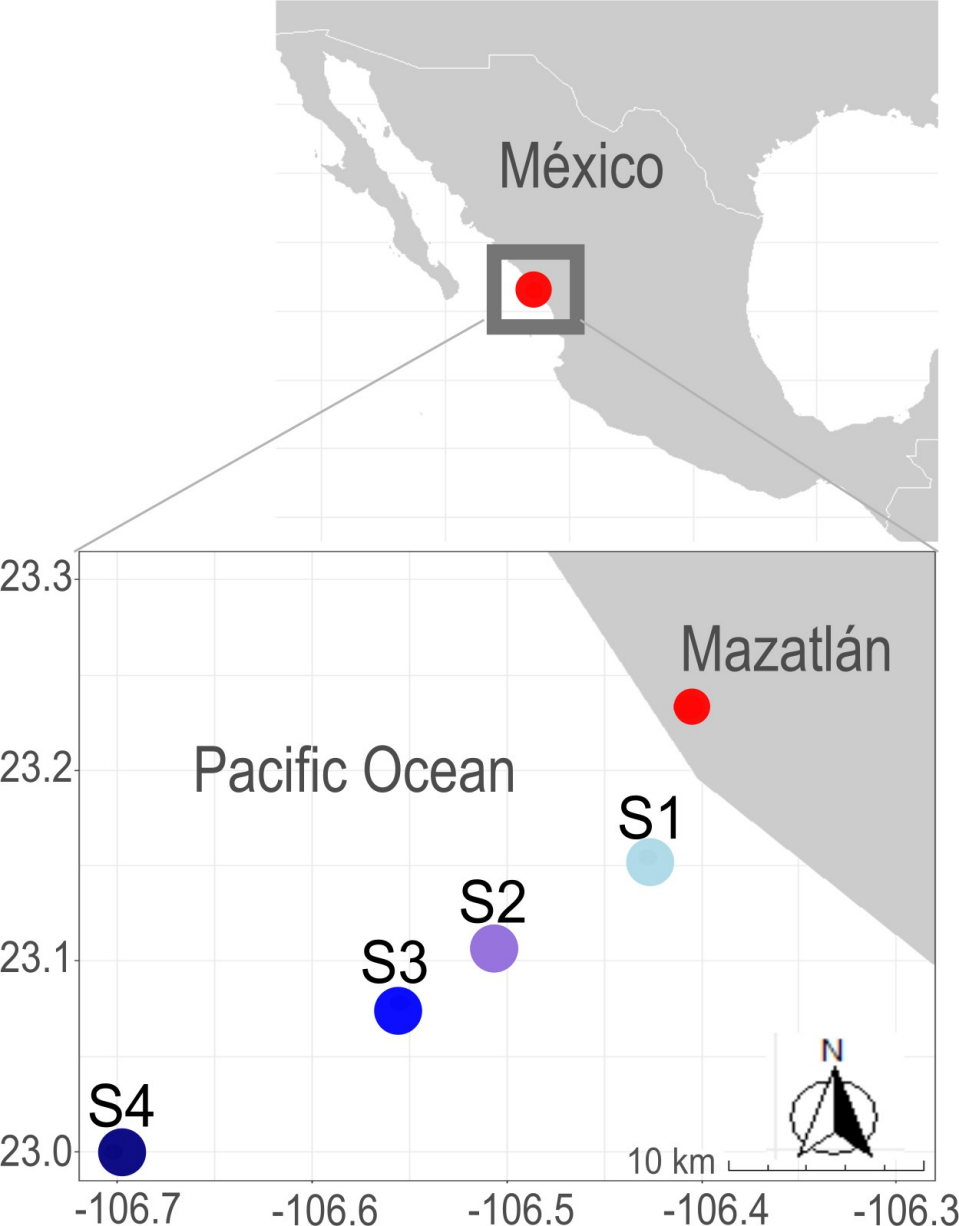
Sampling stations along a transect off the coast of the Port of Mazatlán (cyan = S1, purple = S2, blue = S3, dark blue = S4). The stations nomenclature is indicated in Table 1. Map was generated with the R package ggplot2 v. 3.4.0 [47].

We collected sediment cores along a transect offshore from the Port of Mazatlán (23.2329 N, -106.4062 W), within the OMZ of the Mexican Pacific Ocean (Fig 1) that is part of the Eastern Tropical North Pacific (ETNP). The ETNP comprises the largest OMZ in the world [2] with a functionally anoxic core [42, 43], it is one of the major sites of water column denitrification [44] and a productive area that has recorded one of the highest O_2_ losses in the global ocean [45, 46]. It is important to highlight that sampling permits are not applicable for the collection of sediments in this region.

We collected sediment samples by multicore during the oceanographic cruise “MazV” in April 2019 on board the R/V “El Puma” of the National Autonomous University of Mexico (UNAM) in four stations along a bathymetric gradient (from the coast and extending to 36 km offshore; Table 1). Onboard, we aseptically obtained subsamples from the surface (0–10 cm) and the subsurface (10–20 cm) cores, except for station S2 that was only recovered superficially because of sampling difficulties given the presence of solid rock. We stored the sediment subsamples in sterile polypropylene 15 mL tubes at -80°C in absolute dark until processed in the laboratory (within the next 48 h) for DNA extraction. To analyze pH and organic carbon (C), we used a dry fraction of the sediment subsamples. We measured pH in a sediment:water ratio of 1:2.5 with a pH meter (Hanna Mod. HI2020-01). To estimate organic C, we used the modified Walkley-Black method, which consists of exothermic heating and oxidation with potassium dichromate and sulfuric acid [48]. Overlying the sediment cores from each station, we measured the concentration of dissolved oxygen (DO), temperature, and salinity with a conductivity-temperature-depth (CTD; SeaBird 19 plus) profiler coupled to a rosette on the R/V “El Puma” (S1 Table). As DO, temperature, and salinity were only measured in the water overlying surface sediments, we suggest considering their measurement in subsurface sediments for subsequent studies.

**Table 1:** Stations where sediment subsamples were collected in the OMZ of the Mexican Pacific off Mazatlán. Subsample labels denote: surface (.0), and subsurface sediments that were collected at a 20 cm-depth (.20).

Stations	Subsamples	Latitude	Longitude	Depth (m)	Distance from the coast (km)
S1	S1.0, S1.20	23° 9’ 15.90"	-106° 25’ 40.38"	32	4
S2	S2.0	23° 6’ 27.42"	-106° 30’ 19.38"	75	13
S3	S3.0, S3.20	23° 4’ 40.80"	-106° 33’ 19.02"	105	19
S4	S4.0, S4.20	23° 0’ 0"	-106° 42’ 7.98"	705	36

### 2.2. DNA Extraction, amplification, and sequencing

We obtained environmental DNA for each subsample (0.25 g) employing the DNeasy PowerSoil kit (Qiagen, Carlsbad, CA, United States) and following the guidelines provided by the manufacturer. To quantify the DNA, we used a Qubit® 2.0 Fluorometer (Invitrogen by Life Technologies). The Genomic Services Laboratory (LANGEBIO, Irapuato, Mexico) performed Illumina MiSeq paired-end (2 × 300) sequencing, targeting the ITS1 of the ribosomal RNA gene cluster with the primers set ITS1F (5 ’-CTTGGTCATTTAGAGGAAGTAA-3‘) and ITS2 (5 ’-GCTGCGTTCTTCATCGATGC-3’; [49, 50]). As a result, we yielded to around 25,000 reads per subsample. The dataset is available at NCBI Sequence Read Archive under the BioProject **PRJNA822656**.

### 2.3. Bioinformatics analyses

We processed Illumina raw reads with the ITS-specific variation DADA2 v. 1.18.0 [51] of the R statistic package [52]. Briefly, we inspected read quality profiles, removed ambiguous bases (maxN = 0) with <1 expected errors (maxEE = c(1,1)), and then removed primer and adapter sequences with Cutadapt [53]. Next, we inferred the ASV for each subsample considering the specific error rates based on quality scores. To assemble paired-end reads, we considered 50 pb as minimum overlap without allowing mismatches. To remove chimeras, we used the DADA2 function “removeBimeraDenovo”. To assign taxonomy to the ASV, we used the trained classifier IDTAXA [54] of DECIPHER v. 2.18.1 Bioconductor package [55] against the February 2020 update of the eukaryotic database UNITE [56]. We filtered the ITS dataset to keep solely fungal reads. To avoid overestimation of ASV at the species level, we reclustered these assignments using CD-HIT v. 4.7 program [57] with a threshold of 98% [58]. Lastly, we assessed the sampling effort from rarefaction and extrapolation curves with the R package iNEXT v. 2.0.20 [59].

### 2.4. Composition and diversity analyses

To depict the relative abundance and shared taxa among subsamples, we used the R packages ggplot2 [47], complexHeatmap [60], and UpSetR [61]. To evaluate alpha diversity, we estimated the rarefied Shannon diversity using iNEXT v. 2.0.20 [59] along with bootstrap 95% confidence intervals (1,000 replicates), and the Pielou evenness using the R package “vegan” v. 2.5–7 [62]. The heatmaps combined with dendrograms of hierarchical agglomerative clustering (based on Bray-Curtis dissimilarity matrix) showed the clustering of fungi by subsample according to their relative abundance. We estimated the differences in beta diversity across subsamples through the Bray-Curtis (abundance-based) and Jaccard (presence-absence) dissimilarities. To draw each dissimilarity matrix, we employed the Ward´s clustering method. To represent community dissimilarities among subsamples in terms of the environmental setting, we performed Constrained Correspondence Analysis (CCA) using the “ordistep” function (from the “vegan” package), which performed a stepwise selection of explanatory variables.

### 2.5. Functional guilds

The accurate assignation of trophic strategies to taxonomic units remains as a major challenge in mycology. Nonetheless, fungal functional categorization could represent a useful guide to navigate the effect of functional diversity on ecosystem processes [63]. The python-based tool, FUNGuild, resolves fungal functional guilds based on taxonomic affinity [64]. So, we implemented this approach (FUNGuild v. 1.1 database; https://github.com/UMNFuN/FUNGuild) to assign trophic modes to our ASV. It should be noted that precise community-wide conclusions are still unattainable since: 1) many genera comprise several trophic strategies; 2) guild data is insufficient for many fungal groups [65]; and 3) the lifestyles of marine fungal taxa remain largely unknown. However, this invaluable information should pave the way for further improved work on deep-sea fungi.

## 3. Results

### 3.1. Environmental conditions

The concentration of dissolved O_2_ at the bottom of the water column (overlying the sediments) decreased towards the open ocean ranging from 208 μmol kg^-1^ (coastal station S1) to 0.97 μmol kg^-1^ (offshore station S4). Exclusively, the S1 (208 μmol kg^-1^) exceeded the dissolved O_2_ concentration of the OMZ (<22 μmol kg^-1^; [1]), probably due to the mixing of adjacent ocean currents during sampling. Salinity and temperature followed the same pattern as dissolved O_2_, ranging from 35 PSU (S1) to 34.53 PSU (S4), and 22.2°C (S1) to 6.6°C (S4), respectively. Regarding sediment subsamples, pH was higher in the subsurface subsamples of stations S1 and S4, ranging from 7.5 (subsample S4.0) to 8.3 (subsample S1.20). Likewise, the organic C pattern of was higher in the subsurface subsamples of the stations S1 and S4, showing an increase towards the open ocean ranging from 0.86 (subsample S1.0) to 8.2 (subsample S4.20; S1 Table).

### 3.2. Fungal community composition

After filtering and denoising processes, 313,294 ITS1 assembled sequences were obtained (S2 Table). All rarefaction and extrapolation curves in the sediment subsamples reached the asymptote (S1 Fig), indicating that the sequencing depth was adequate. We inferred a total of 518 fungal ASV (151,925 sequences), which were clustered into 353 ASV at the species level after the CD-HIT analysis (S3 Table).

**Fig 2:**
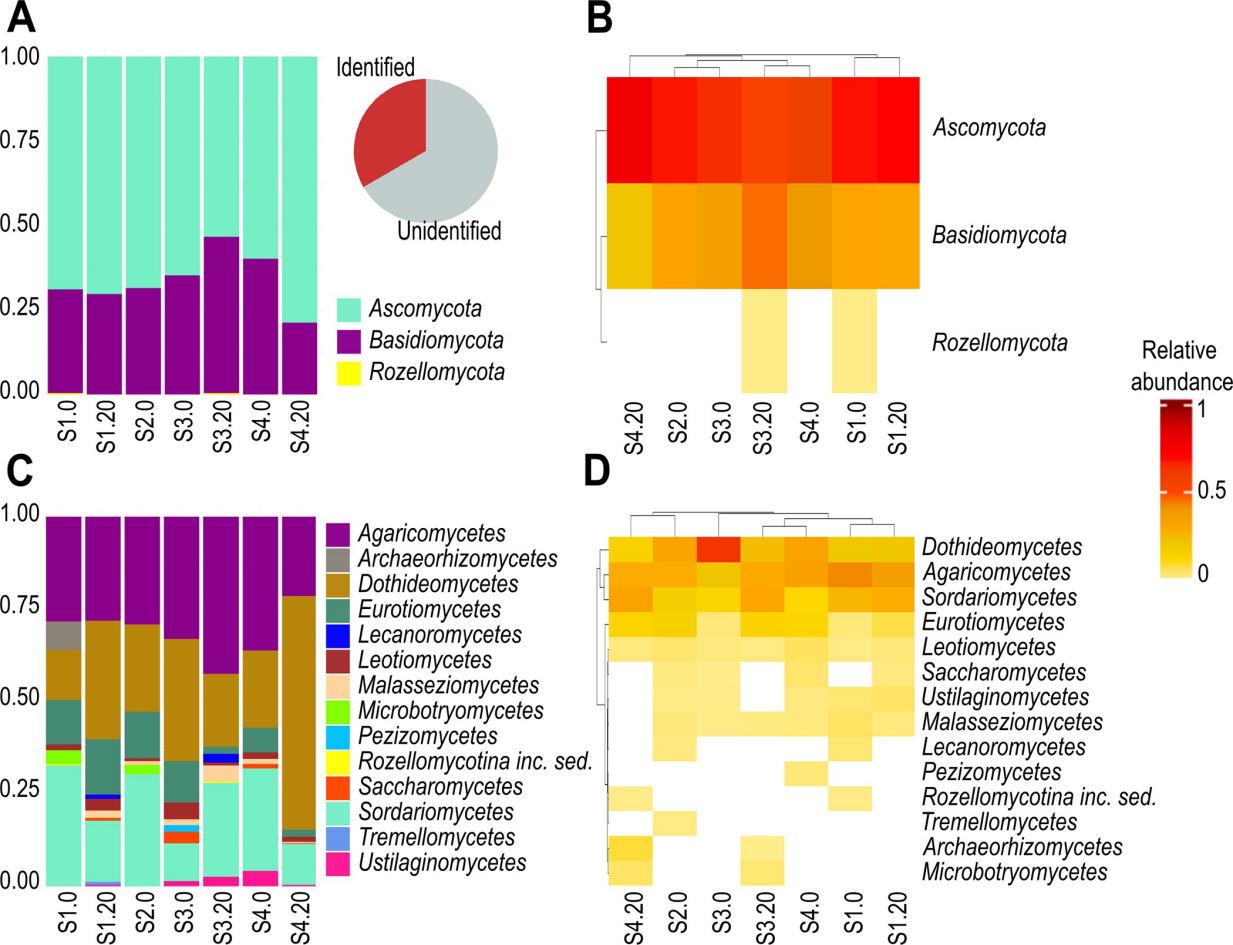
Relative abundance of fungal phyla and classes across subsamples. Stacked bar plots depicted the fungal relative abundances at the phylum (A) and class (C) level of the identified taxa across subsamples. Clustered heatmaps showed the fungal richness (horizontal lines in each subsample), and their relative abundance (white-red coded lines) at the phylum (B) and class (D) level across subsamples. The subsamples nomenclature is indicated in Table 1.

The ASV belonged to three phyla, 14 classes, 33 orders, 69 families, and 80 genera. The *Ascomycota* was the most abundant phylum among all sediment subsamples, accounting for 236 ASV (73.21% of the relative abundance of identified sequences) with *Dothideomycetes* as the dominant class (46.87% of identified sequences), followed by *Sordariomycetes* (14.91%) and *Eurotiomycetes* (4.80%). The *Basidiomycota* was the second most abundant phylum with 79 ASV (26.75%), with *Agaricomycetes* (24.75%) as the most abundant class, followed by *Malasseziomycetes* (1.03%), and *Ustilaginomycetes* (0.67%). The basal clade *Rozellomycota* was the least abundant with 2 ASV (0.04% of identified sequences). Furthermore, 69.01% of the relative abundance of all sequences corresponded to unidentified phyla. Both *Ascomycota* and *Basidiomycota* were detected in all the subsamples varying in their relative abundance, yet *Rozellomycota* was detected only in subsamples S1.0 and S3.20 (Fig 2).

The top abundant families were *Didymosphaeriaceae* (31.93% of the relative abundance of identified sequences), *Lycoperdaceae* (7.29%), *Agaricaceae* (5.96%), *Stachybotryaceae* (4.79%), *Aspergillaceae* (3.84%), *Xylariaceae* (3.66%), *Periconiaceae* (3.57%), and *Hypocreaceae* (2.36%). At the genus level, the top abundant genera were *Paraphaeosphaeria* (29.98% of identified sequences), *Agaricus* (5.96%), *Periconia* (3.57%), *Lycoperdon* (2.75%), *Penicillium* (2.71%), *Trichoderma* (2.36%), and *Hypoxylon* (2.16%). This information is presented in S3 Table.

**Fig 3:**
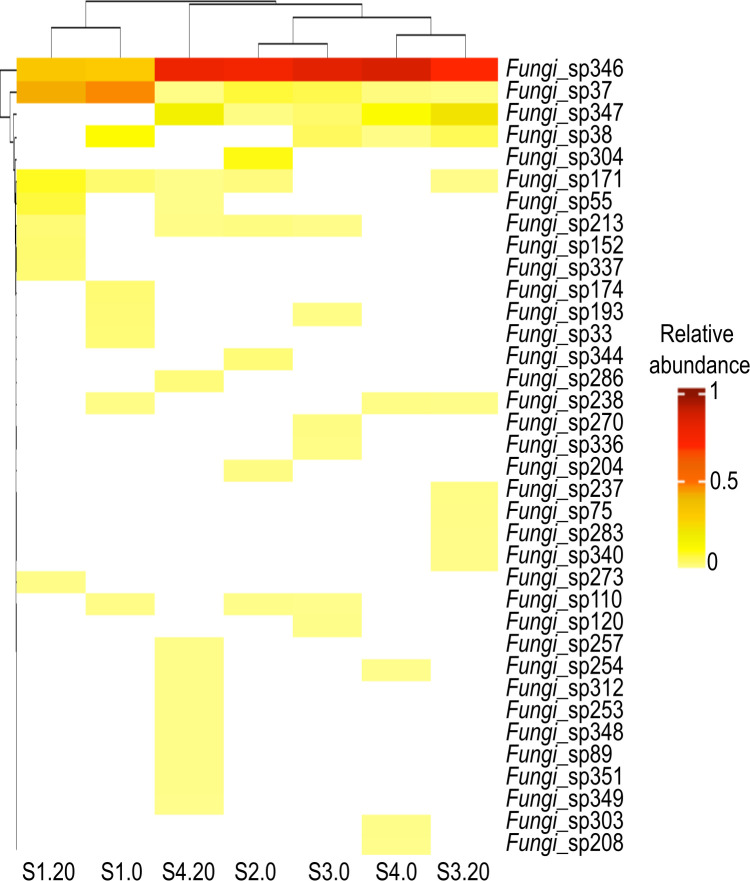
Relative abundance of *Fungi* spp. across subsamples. Clustered heatmap showed the richness of fungi that were taxonomically assigned solely at the kingdom level as *Fungi* spp. (horizontal lines in each subsample) and their relative abundance (white-red coded lines) across subsamples. The subsamples nomenclature is indicated in Table 1.

At the species level, 36 fungal ASV (accounting for 69.01% of the relative abundance of all sequences) could only be taxonomically assigned to kingdom level as *Fungi* spp., with two being dominant: *Fungi* sp. 346 (54.53%) and *Fungi* sp. 347 (10.14%), accounting for 64.67% of all sequences (Fig 3). Dominant ASV also included *Paraphaeosphaeria* sp. 130 (4.29% of all sequences), *Paraphaeosphaeria angularis* (1.63%), *Fungi* sp. 37 (1.50%), *Paraphaeosphaeria michotii* (1.48%), *Paraphaeosphaeria* sp. 136 (1.46%), *Fungi* sp. 286 (0.72%), and *Ascomycota* sp. 226 (0.67%). Remarkably, 285 ASV (9.01% of all sequences) were rare taxa (<0.1% relative abundance each).

### 3.3. Alpha and beta diversity patterns

**Fig 4:**
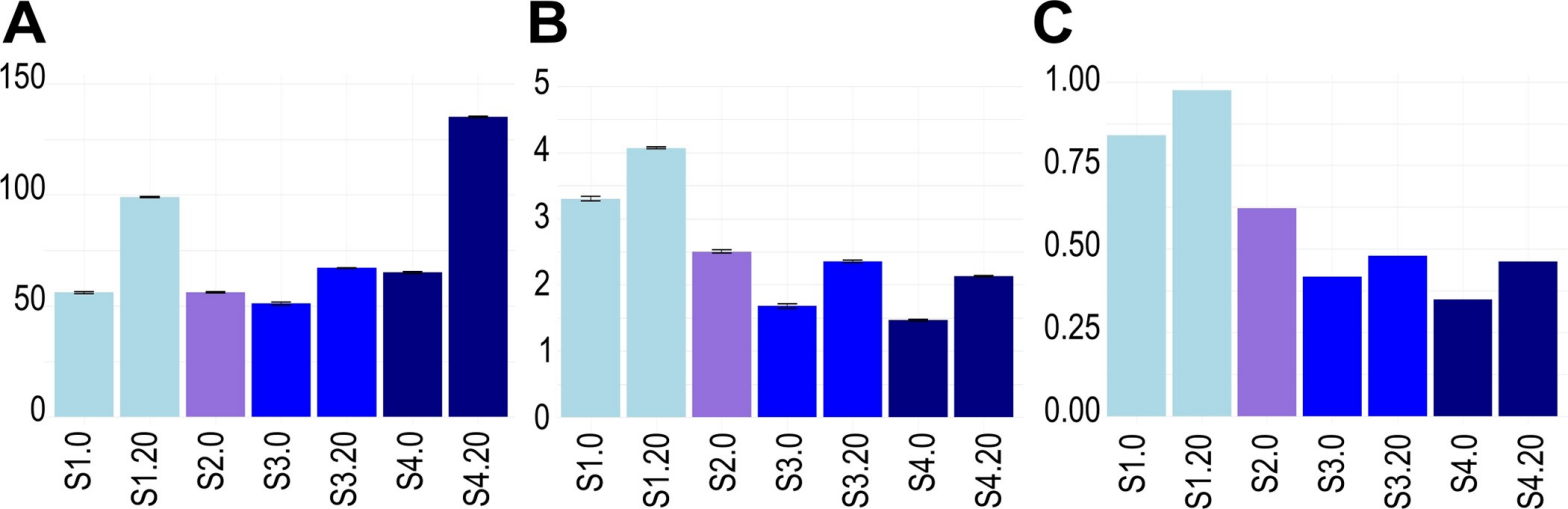
Alpha diversity estimates of fungal ASV in sediment subsamples. Bar plots depicting the richness of ASV at the species level (A), Shannon index (B), and evenness (C). The error bars indicate the standard deviation of richness and the Shannon index. The color of each bar corresponds to its station (cyan = S1, purple = S2, blue = S3, dark blue = S4). The subsamples nomenclature is indicated in Table 1.

Richness ranged from 51 (subsample S3.0) to 134 (subsample S4.20) ASV, Shannon diversity index ranged from 1.47 (subsample S4.0) to 4.07 (subsample S1.20), and the evenness ranged from 0.35 (subsample S4.0) to 0.97 (subsample S1.20). Both Shannon index and evenness showed the highest values in nearshore stations and subsurface subsamples (Fig 4 and S4 Table).

**Fig 5:**
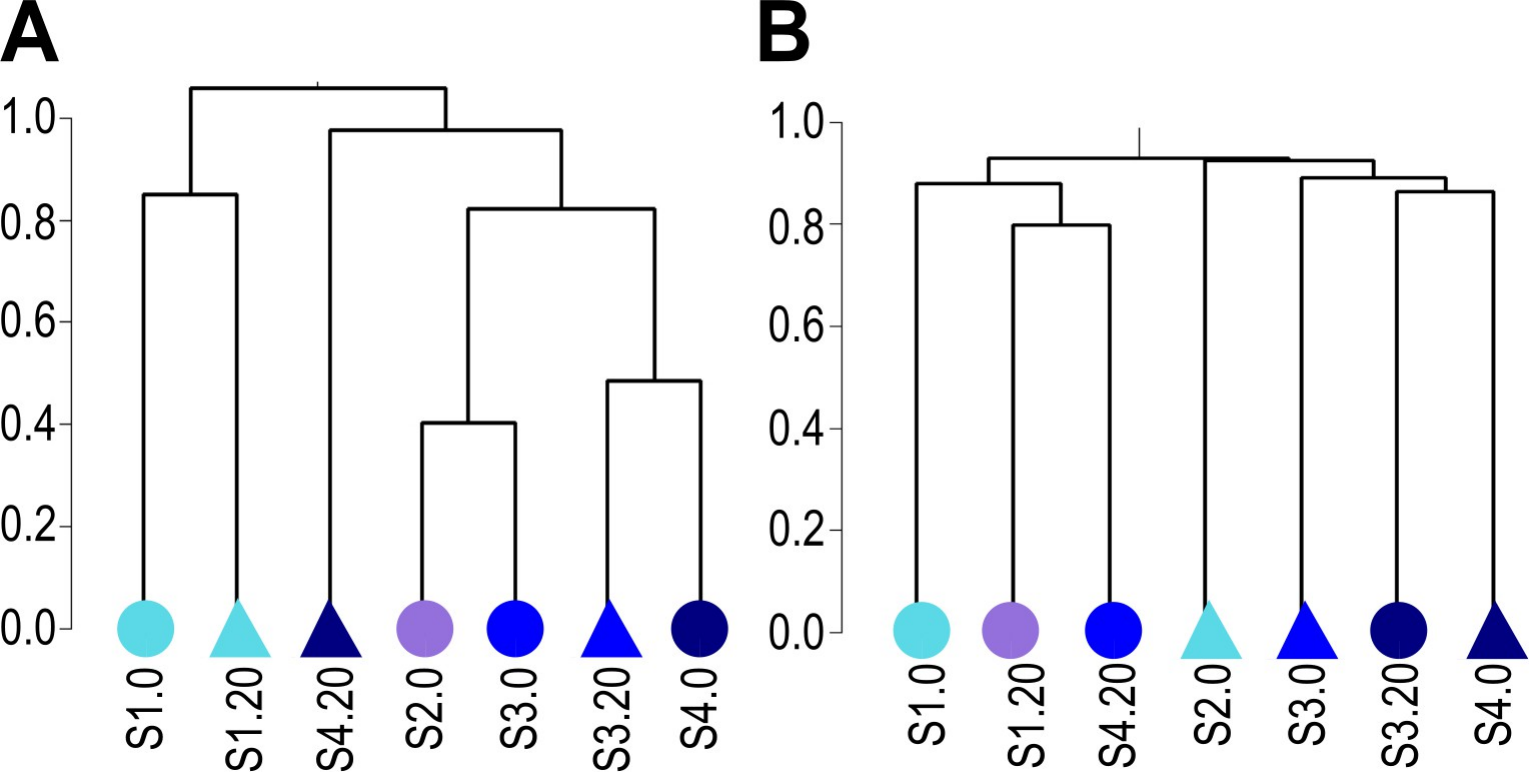
Beta diversity estimates of fungal ASV in sediment subsamples. Dendrogram based on Bray-Curtis (A) and Jaccard (B) metrics indicating dissimilarities of fungal communities across sediment subsamples, where height (*y-*axis) indicates distance given the dissimilarity metrics. The color of each subsample corresponds to its station (cyan = S1, purple = S2, blue = S3, dark blue = S4). Circles represent surface subsamples and triangles represent subsurface subsamples. The subsamples nomenclature is indicated in Table 1.

The Bray-Curtis dissimilarity showed a high variation among the subsamples, with distances fluctuating between 0.40 (S2.0 and S3.0), and 0.98 (S1.0 and S4.20). The clustering patterns were detected in accordance with the distance from the coast (nearshore *vs* offshore). The Jaccard index showed community clustering based on presence-absence data, varying between 0.80 (in subsamples S2.0 and S3.0) and 0.92 (in subsamples S1.20 and S3.0). The clustering patterns were detected in accordance with the depth in sediment (0 cm *vs* 20 cm; Fig 5 and S4 Table).

**Fig 6:**
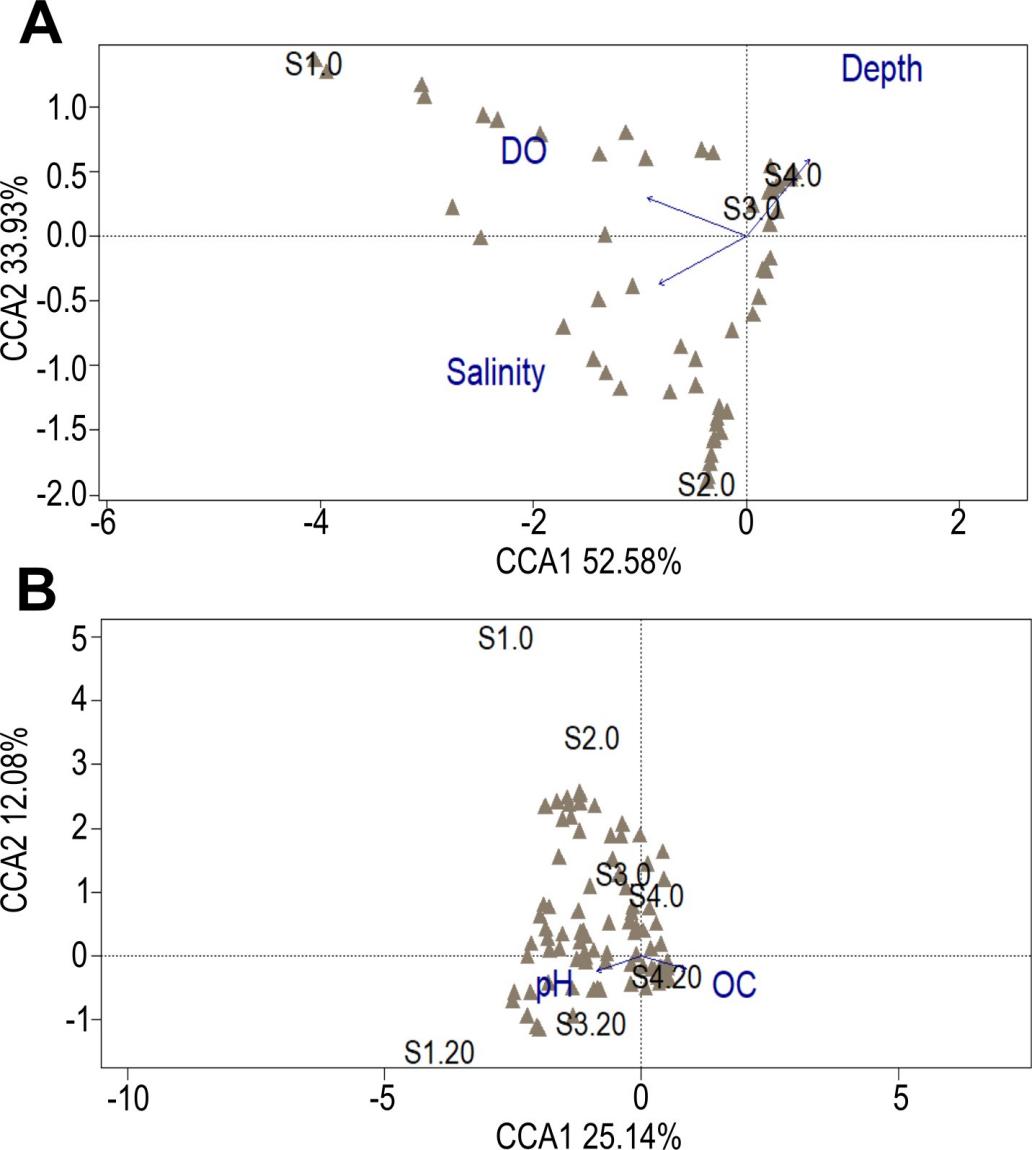
Constrained Correspondence Analysis plots of fungal ASV associated with environmental variables. The variables were measured at the bottom of the water column (A) and in the sediments (B) along the bathymetric transect. The percent of the variation in the fungal community explained by each axis is indicated in parentheses after the axis label. DO, dissolved oxygen; OC, organic carbon. Fungal ASV are indicated as gray triangles. The subsamples nomenclature is indicated in Table 1.

According to the CCA, depth, DO and salinity in the bottom water explained 86.51% of the variation in the fungal community, with ASV from the surface subsamples further offshore, S4.0 and S3.0, perhaps associated with depth. In addition, the organic C and pH showed a weak association (explaining 37.22% of the variance) with the fungal community. The surface subsamples (especially the nearshore ones) were the least associated with these variables, whereas the ASV from the subsurface subsample S4.20, only showed a slightly higher association with organic matter (Fig 6).

**Fig 7:**
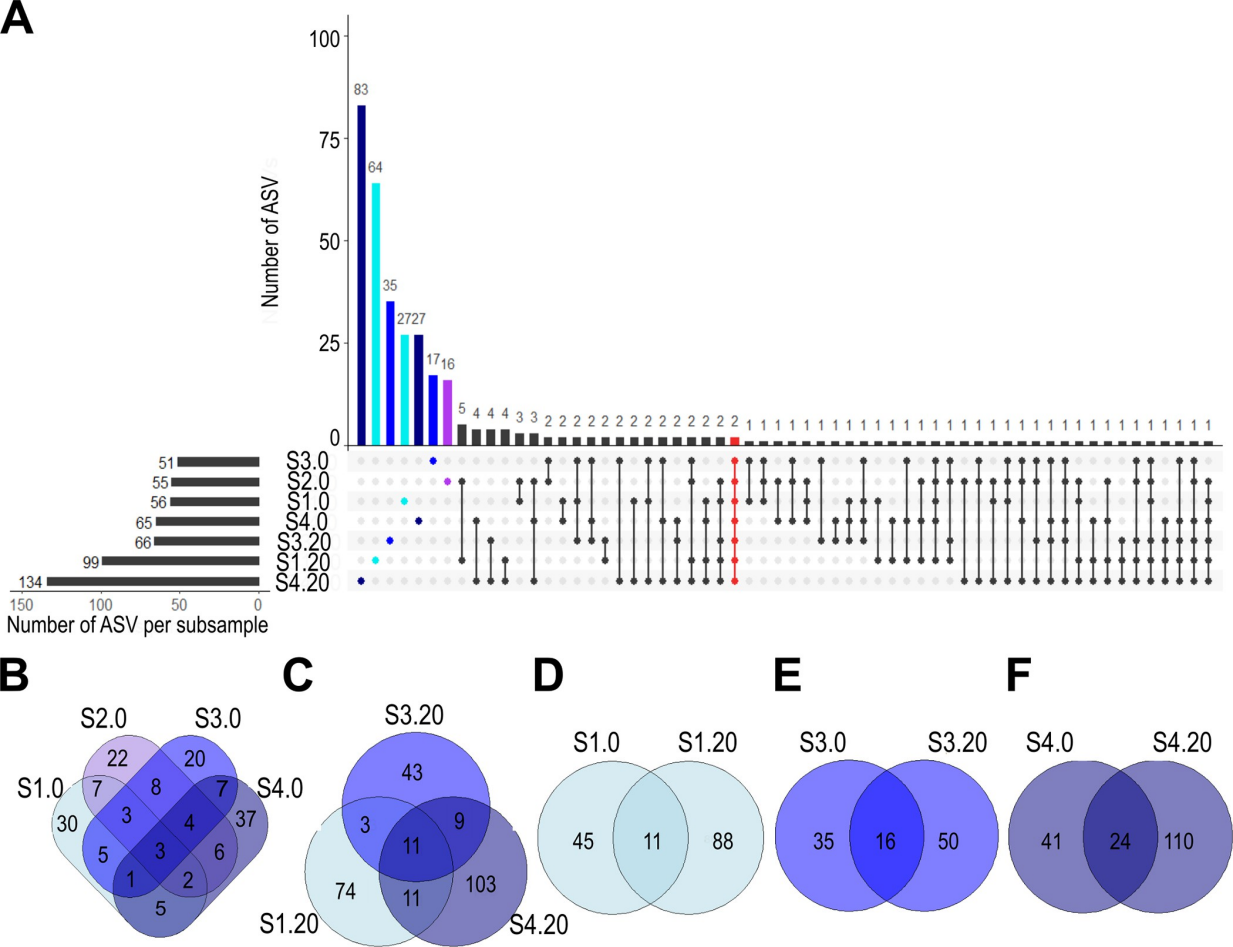
Upset plot and Venn diagrams. Upset plot representing unique (cyan = S1, purple = S2, blue = S3, dark blue = S4; station nomenclature in Table 1) and common (highlighted in red) fungal ASV occurrence across all subsamples stacked in the *x*-axis (A). Dots below a bar depict the occurrence of the ASV for each subsample. Vertical line figures the ASV common to several subsamples. The number of ASV per subsample represents the richness of each one. Besides, Venn diagrams exhibited the unique and common ASV among surface sediment subsamples (B), subsurface subsamples (C), and at different sediment depths at each station (D).

Our results evidenced that only two ASV (from a total of 353 at the species level) were common among the seven subsamples, whilst most of the taxa (269 ASV accounting for 76.20% of all of them) occurred at a single subsample. In addition, only three ASV were present in all surface subsamples, and only 11 in all subsurface subsamples. Within the same station, only 11 ASV were common to S1.0 and S1.20, 16 to S3.0 and S3.20, and 24 to S4.0 and S4.20 (Fig 7).

### 3.4. Functional guilds

**Fig 8:**
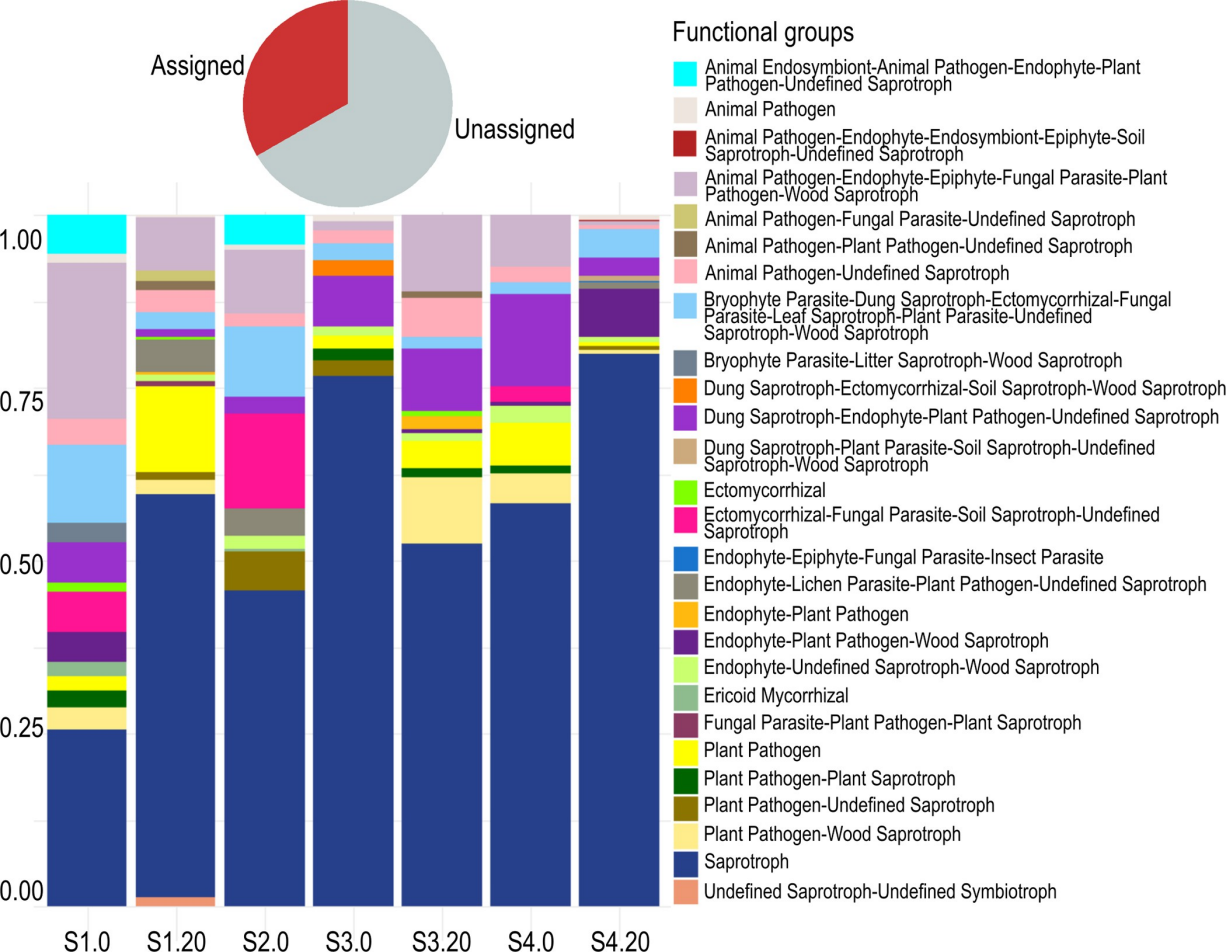
Assigned functional guilds. Stacked bar plot showing the relative proportion (y-axis) of the fungal trophic modes (shown in red in the pie chart) in sediments across subsamples in the eastern tropical Pacific oxygen minimum zone (x-axis). The subsamples nomenclature is indicated in Table 1.

FUNGuild analysis led to the delimitation of 37 trophic modes (re-clustered into 27 excluding the differentiation based on specific substrata; S5 Table), accounting for 22.67% of total reads (Fig 8). This analysis revealed the dominance of saprotrophs in all the sampling sites, particularly in the deepest stations. The richest and most equative distribution of functional diversity was observed near the coast where the presence of pathogenic and parasitic lifestyles were copious (Fig 8).

## 4. Discussion

Even though the Pacific Ocean includes the largest OMZ in the world, its mycobiota remains poorly studied, with major gaps in the Mexican Pacific OMZ. This work presents the first record of fungal community composition and diversity in the sediments off the Port of Mazatlán, reporting 353 ASV and showing characteristic diversity patterns along a bathymetric transect and across two depths in the sediment. Notably, the magnitude of diversity exceeded former figures based on the analysis of the ITS region reported for the water column (237 ASV; [66]) and sediment samples (102 ASV; [22]) of the OMZ of the ETNP. Overall, our results highlight that OMZ off Mazatlán supplies a suitable niche for fungal proliferation that deserves investigation to identify key microbial constituents and to obtain a complete thoughtful of ecosystem functioning.

### 4.1. Taxonomic diversity

The dominance of *Ascomycota* and *Basidiomycota* in all subsamples collected in the Mexican OMZ (Fig 2) resembles previous findings in several marine environments [20, 29, 38, 67, 68]. We also detected a minor proportion of *Rozellomycota*, with a distribution limited to subsamples S1.0 and S3.20. Members of this phylum have been recorded from numerous marine regions [35, 39, 66, 68, 69] and have been recognized as parasites of zoosporic fungi and Oomycota [70].

At the species level, the present study revealed the copious occurrence of unidentified, uncultured phylotypes (36 ASV accounting for 69.01% of the overall reads; Fig 3). Remarkably, *Fungi* sp. 346 and 347 (especially abundant in the deepest subsurface subsample S4.20) showed a 100% homology (query cover = 100) with the phylotype DSF-Group1 based on a BLAST search against the GenBank database (KT758162.1 and KT758149.1 respectively), suggesting their affiliation to this group. This uncultured phylotype has been considered as a globally distributed deep-sea endemic cluster that is probably adapted to O_2_-deficient environments given its high abundance in our subsurface sediments [28, 69, 71–75]. The plethora of unidentified phylotypes in this study indicates that the OMZ sediments in the Mexican Pacific may harbor novel fungal taxa (such as *Fungi* spp. that were only identified at kingdom level). This could be due to the vast majority of fungal species remaining unknown and the marked bias of the ITS-reference database, based on terrestrial representatives, towards these *Basidiomycota* and *Ascomycota* [76]. In this sense, a polyphasic taxonomic approach (*i*.*e*., cultivation-independent studies with cultivation-dependent studies) should aim to understand the mycobiota inhabiting OMZ [77].

Members of the *Paraphaeosphaeria* were copiously represented in our samples (31.30%), especially in the subsurface (S3 Table). These ASV resemble those formerly registered in deep-sea sediments from the South China Sea (GenBank database accession KT758166.1), an OMZ in the Gulf of California (GenBank database BioProject PRJNA793088; [22]), and salt marsh grasses (GenBank database BioProject PRJNA623945; [78]). Considering the board ecological strategies of this genus [79, 80], we assume an ecological niche shift (*sensu* Selosse et al. [81] coupled to possible adaptation to the extreme conditions in OMZ sediments. However, this assumption requires further testing of their active roles under anoxic conditions.

Within *Malasseziomycetes*, *Malassezia*-like fungi occurred in most of the subsamples (Fig 2). Members of this genus distribute across assorted marine habitats [30, 69, 75, 82], occurring as pathogens of marine mammals [83] and in association with marine sponges and deep-sea invertebrates [84]. In our samples, the wide distribution of *M*. *restricta* (S3 Table) agrees with the presence of large populations of benthic polychaetes (*e*.*g*., *Sabellidae* and *Terebellidae* [85]) in the Pacific. This suggests a host-parasite relationship that may influence the benthic community structure of OMZ.

Microorganisms inhabiting OMZ are largely adapted to use nitrates in the absence of oxygen through denitrification processes. Fungi in marine environment play an important role in nutrient cycling [86]. These osmotrophs reduce nitrate or nitrite to nitrous oxide (incomplete denitrification pathway), even under conditions of elevated pressure and low temperature, which are characteristic of the deep-sea. In this regard, *Penicillium* and *Trichoderma* taxa are known for their contribution to the marine nitrogen cycle through the denitrification process [21, 23, 66]. Interestingly, species of these genera showed a high richness in our samples (S3 Table), including denitrifying species such as *Penicillium melinii* [66]. This suggests their role in modeling oxygen-depleted ecosystems, affecting the geo-biochemical landscape in OMZ [25].

### 4.2. Diversity patterns along the bathymetric transect

Highest alpha diversity values and functional heterogeneity were observed in the nearshore subsamples (S1.0 and S1.20; Fig 4). This pattern could be explained by decreases in evenness towards the open ocean due to the dominance of *Fungi* sp. 346 and *Fungi* sp. 347. The higher diversity was obtained nearshore, agreeing with previous research [23, 33, 34]. Furthermore, surface subsamples distributed along the bathymetric transect showed a high turnover of the fungal community composition, with barely three ASV (including *P*. *melinii*, *Fungi* sp. 346 and *Fungi* sp. 347) common among subsamples (Fig 7). In terms of beta diversity, the highest dissimilarity values based on abundance were recorded between the most coastal subsample (S1.0) and the deepest subsurface subsample (S4.20; Fig 5), which indicate that the dissimilarity is not only related to the spatial distance (~32 m; Table 1) between these two stations but is also related to the properties of each depth in the sediment.

The dissolved O_2_ did not show a clear association with the fungal community in offshore subsamples, which may suggest the adaption to oxygen-depleted environments. Additionally, the depth in the water column was associated with the fungal community composition from S3.0 and S4.0 open-ocean subsamples (Fig 6). This distribution pattern agrees with that registered in the study on the global biogeography of marine fungi [36] and the fungal communities along the Western Pacific Ocean [67]. This could be linked to distinct depth-related nutrient conditions because allochthonous (*e*.*g*., from terrestrial and atmospheric sources) and autochthonous (*e*.*g*., upwelling of nutrient-rich water) input of organic matter (in accordance to the FUNGuild analysis revealing a large proportion of saprotrophs) is common in or near organic-rich coastal areas, while is less frequent in the organic-poor oligotrophic open ocean. In open ocean only those organisms (such as fungi) able to utilize the remnant refractory organic matter deposits (saprobes) and pathogens can thrive.

### 4.3. Vertical diversity patterns in the sediment

Subsurface subsamples presented higher alpha diversity values than those from the surface (Fig 4), even though previous investigations (mainly large spatial scale studies with bacteria; [87]) have recorded lower abundances of marine microbes with increasing depth and age of marine sediments [88, 89]. Additionally, similar to surface sediments, the subsurface sediments along the bathymetric transect showed high fungal community turnover, with solely 11 ASV common among each other. Notably, *Rousella solanni* was the only ASV that was not present in the surface sediments (Fig 7), thus it could be adapted to the oxygen-depleted conditions of the sediments collected at 20 cm depth.

Our findings at the small-scale (0 *vs* 20 cm sediment depth) revealed a heterogeneous fungal community assemblage, with most ASV (76.20%) restricted to a single subsample (Fig 7). These compositional differences have also been registered in the Peru Margin and Peru Trench [30], Central Indian Basin [41], Canterbury Basin [38], and South China Sea [32], hinting at the differential occupation of vertical microniches in the sediment by fungi. In this context, environmental filtering observed in bacteria [90–92] may influence the establishment of some fungal ASV according to the niche requirements of the species. Thus, the diversity pattern obtained may be related to the slightly higher percentage of organic C in the subsurface sediments (Fig 6), as has been suggested for the marine subsurface biosphere [40, 93] and benthic deep-sea environments [33, 94].

## 5. Conclusions

Our results revealed high fungal diversity in the OMZ of the eastern tropical Pacific, with the *Ascomycota* and uncultured, unidentified phylotypes as dominant elements. The highest diversity levels were recorded in nearshore subsamples along the bathymetric transect and in subsurface subsamples across the vertical pattern. We suggest that *Fungi* sp. 346 and 347, *P*. *angularis*, *R*. *solanni*, and *P*. *melinii* identified in this OMZ could be key microbial players in carbon and nitrogen cycles. In addition, we highlight the copious abundance of fungi in anoxic subsurface sediments, which could be essential for marine ecosystem functioning given the extent of the subsurface biosphere. Furthermore, our results indicated high heterogeneity of fungal composition at a small spatial scale, which may suggest its dependence on the biogeochemical conditions of each vertical microniche in the sediment. Hence, this work provides baseline information on the high fungal diversity in the OMZ sediments despite their extreme conditions.

## Supporting information

S1 Fig Rarefaction curves of fungal ASV, where all curves reached the plateau. The subsamples nomenclature is indicated in Table 1.
(TIF)

S1 Table Environmental variables in each sampling station. (XLSX)

S2 Table Summary statistics of the number of reads through the analysis pipeline. (XLSX)

S3 Table Number of fungal ASV reads from sediment subsamples collected in the oxygen minimum zone of the Pacific Ocean. (XLSX)

S4 Table Alpha and beta diversity estimates. (XLSX)

S5 Table Fungal functional assignation. (XLSX)
