# Supplementary figures and images for: Fungal diversity in sediments of the eastern tropical Pacific oxygen minimum zone revealed by metabarcoding
Source: PLoS One. 2024 May 13;19(5):e0301605. doi: 10.1371/journal.pone.0301605 (PMC11090300; doi:10.1371/journal.pone.0301605)

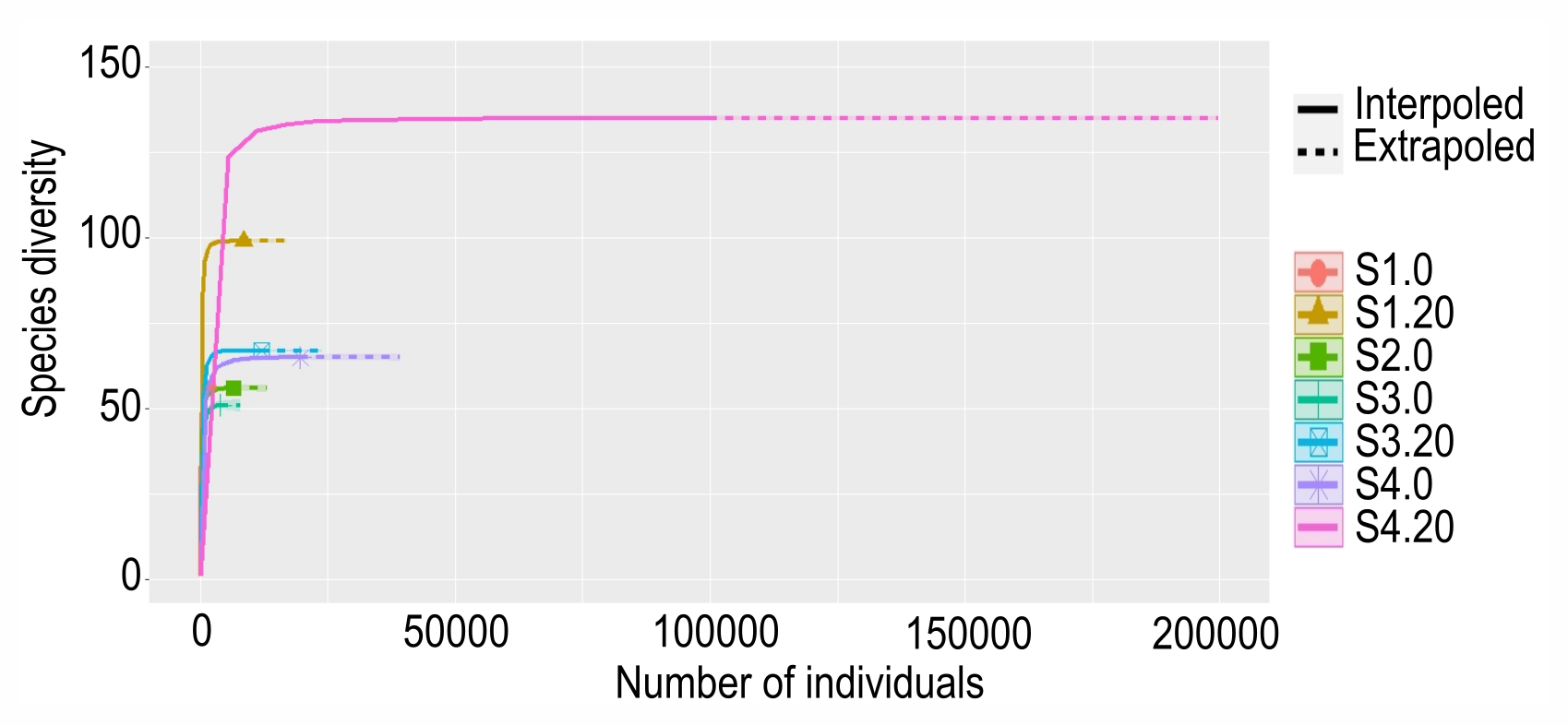

Supplement: S1 Fig — The subsamples nomenclature is indicated in Table 1. (TIF) [file pone.0301605.s001.tif]
